# Fibroepithelial polyp of the prepuce: A rare complication of long-term condom catheter usage

**DOI:** 10.4103/0970-1591.40628

**Published:** 2008

**Authors:** John S. Banerji, Sanjeev Shah, Nitin S. Kekre

**Affiliations:** Department of Urology, Christian Medical College, Vellore, Tamil Nadu, India; 1 Department of Pathology, Christian Medical College, Vellore, Tamil Nadu, India

**Keywords:** Condom catheter, fibroepithelial polyp, perivascular hyalinization

## Abstract

External urinary drainage devices are in wide clinical uses. There are only a few reports of complications from improper use of condom catheters. We present a case of fibroepithelial polyp of the penis, due to long-term usage of condom catheter. The lesion affected the ventral aspect of the penis. He was successfully treated with wide local excision. The histopathological diagnosis was a fibroepithelial polyp.

## INTRODUCTION

A fibroepithelial polyp of the penis is a rarity, which is strongly linked to long-term, improper condom catheter usage.[1,2] Complications arising from continuous condom catheter usage range from ulceration and necrosis to more severe urethrocutaneous fistulae and urethral diverticulum.[3–5] We describe the fibroepithelial polyp of the prepuce and present it for its rarity.

## CASE REPORT

**Figure 1:**
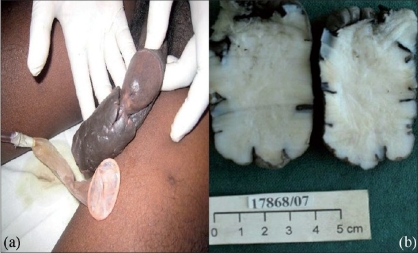
(a) and (b) Lateral aspect of penis with condom catheter, cut section of excised specimen

A 42-year-old man had a traumatic paraplegia 15 years ago. He had bladder incontinence and was initially on clean intermittent catheterization (CIC). For 12 years, however, he has been using a condom catheter drainage system. He presented to the Urology Department with progressively increasing, painless penile lesion for the past 10 years. The lesion was 8 cm in size on the ventral aspect of the penis, confined to the prepuce. This was warty and firm in consistency [Figure 1a]. There was no inguinal lymphadenopathy. There was no communication with the urethra. He underwent a wide local excision under regional anesthesia, after placing a urethral catheter. The excised mass was 8 cm × 5 cm [Figure 1b]. The penile skin was closed with 4-0 chromic catgut sutures. The biopsy was reported to be fibroepithelial polyp of the prepuce. The patient was taught clean intermittent catheterization following catheter removal.

### Histopathology

**Figure 2:**
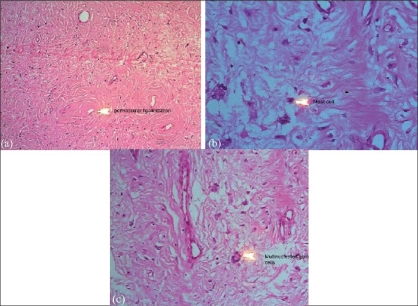
(a-c) Perivascular hyalinization, mast cell, and multinucleate giant cell

There were loosely arranged spindle to stellate cells in a collagenous and edematous stroma. Many variable-sized thin-walled vascular channels, with perivascular hyalinization [Figure 2a] lymphocytes and a few mast cells [Figure 2b] were present within the stroma. Few multinucleate giant cells [Figure 2c] were also present. The overlying stratified squamous epithelium showed acanthosis and hyperkeratosis. There was no evidence of malignancy.

## DISCUSSION

Spinal cord injury and organic mental illness are common causes of urinary incontinence. Condom catheters are considered superior to indwelling catheter drainage. These external drainage devices are simple in concept and design. However, improper fitting and lack of routine hygiene can cause maceration, ulceration necrosis, and even gangrene of the penis or prepuce.

Fibroepithelial polyp of the penis is a rare complication with about only eight reported cases in the literature. Most of these patients have a long-term history of usage of condom catheter. Only one patient in a series by Fetsch *et al*.[1] developed this with long-standing neglected paraphimosis.

The possible pathogenesis is a chronic irritation due to urine leak around an ill-fitting device, leading to maceration, ulceration, and subsequently causing the appearance of the polypoid masses. Although generalized penile edema is a well recognized complication of condom catheter use (secondary to either an allergic reaction of mechanical constriction from the roller ring or adhesive band), localized edema appears to be a less common event. In addition, they could go on for several years, as a lot of these patients are paraplegic with impaired sensations. Although the pathogenesis of this process is unclear, one of Fetsch *et al*.'s[1] patient stated that negative pressure in his condom catheter apparatus occasionally caused skin on the ventral surface of the penis to be sucked into the (rigid collecting) tube. The strong link with condom catheter usage causes us to believe that the basic process is probably a reactive hyperplasia, rather than a true neoplasm.

Fibroepithelial polyps has also been described in the vagina, however, these have a myxoid matrix and their stromal cells have expression of smooth muscle actin and desmin.[6,7]

Fibroepithelial polyp of prepuce differ from conventional cutanous fibroepithelial polyp (also known as a skin tag, acrochordon, soft fibroma, fibroma molle, and fibroma pendulans) by being larger, having notable stromal edema and vascular dilatation and by having greater stromal cellularity. Skin tags are usually < 5 mm in size and they have a predilection for the axilla, neck, and eyelid.

These fibroepithelial polyps could recur after initial excision, especially if the patient continues to use the condom catheter. Hence, it is imperative to suggest alternative methods of urinary drainage like clean intermittent catheterization.
